# Traumatic penile partial amputation caused by rubber band a case report

**DOI:** 10.1016/j.ijscr.2021.106358

**Published:** 2021-08-31

**Authors:** Jason Liarto, Kuncoro Adi

**Affiliations:** Division of Urology, Faculty of Medicine University of Padjadjaran, Hasan Sadikin Hospital Bandung, Jalan Pasteur no 38, Bandung 40161, Indonesia

**Keywords:** Penile amputation, Anastomosis, Case report, Urethra

## Abstract

**Introduction and importance:**

Traumatic amputation of the penis is a rare surgical emergency. Penile amputation is usually caused by self-mutilation, accidents, circumcision, assault and animal attacks. Accidental injury covers a large portion of external genitalia trauma because of its high prevalence and severity of this disease. Here, we report the case of a 21-year-old man who underwent replantation of his self-inflicted partial amputated penis.

**Case presentation:**

We report a case of traumatic penile partial amputation in a 21-year-old man with a history of mental retardation that presented with a one-day history of pain on the penile shaft due to tied penile shaft with a rubber band ten days prior. Genitalia examination showed a partial amputation at the penis shaft region. Reconstruction microsurgery and debridement on the penile shaft and urethral anastomosis were performed. This case highlights the management of traumatic penile partial amputation. The urethral anastomosis and penile replantation were successfully done.

**Clinical discussion:**

Penile amputation is a rare urological emergency. Most of the cases reported with self-mutilation are a result of severe substance-induced psychosis or underlying psychiatric disorder. Factors that contribute to the successful penile replantation include the severity of the penile injury or amputation, type and mechanism of injury, team expertise available, duration of ischemia time, and use of a microscope at the time of neurovascular bundle repair.

**Conclusion:**

A traumatic penile partial amputation is a rare urologic emergency. Self-inflicted amputation is often found in a patient with a history of psychological or mental illness. The limited data on detailed best surgical measures and outcomes is still a concern.

## Highlights

• Penile amputation is a rare urologic emergency.
• Male is prone to have external genitalia trauma more frequently than female.
• The more distal the penile injuries are, the more technically difficult it is to repair.

## 1 Introduction and importance

Traumatic amputation of the penis is a rare surgical emergency [1]. The main etiologies for penile amputation are self-mutilation, accidents, circumcision, assault, and animal attacks [2]. Accidental injury covers a large portion of external genitalia trauma because of the high prevalence and severity of this disease. Penile amputation involves the complete or partial severing of the penis. A complete transection comprises severing of both corpora cavernosa and the urethra. Amputation of the penis may be accidental but is often self-inflicted, especially during psychotic episodes in individuals with mental illness [3].

Herein, we report the case of a 21-year-old man who underwent replantation of his self-inflicted partial amputated penis. This case report has been written in line with the SCARE Criteria [4].

## 2 Case presentation

**Fig. 1:**
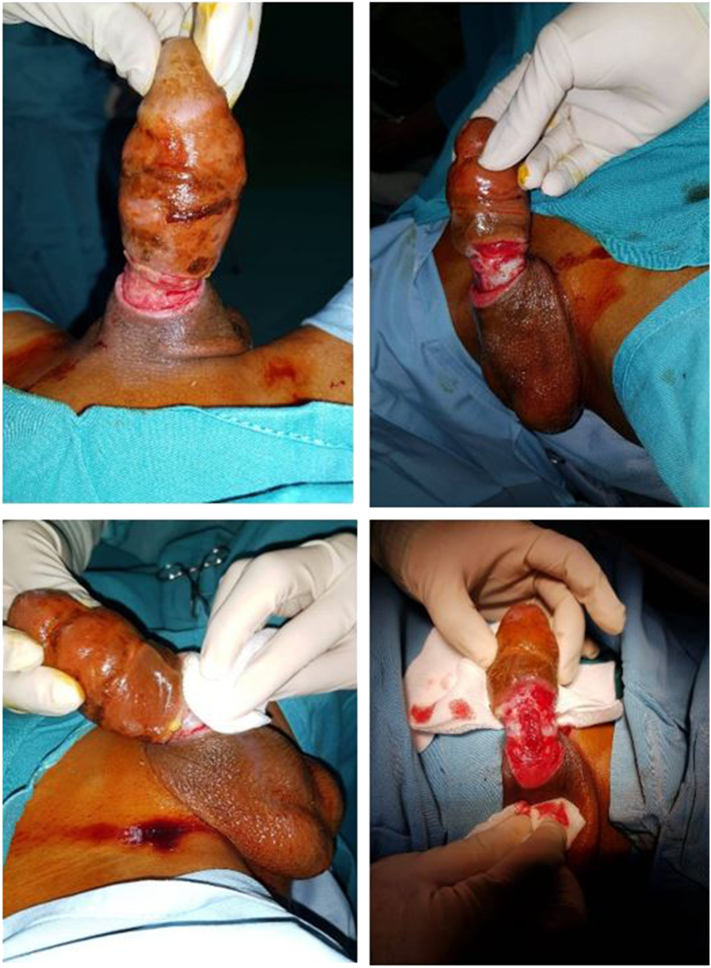
Clinical presentation of the penile shaft showing a traumatic partial amputation on proximal penile shaft.

We report a case of traumatic penile partial amputation in a 21-year-old man who presented a one-day history of painfulness on the penis shaft (Fig. 1). Ten days prior, the patient stated that he tied his penis with a rubber band. One day before admission, the shaft started to expel a foul smell. When examined by his parents, the shaft was still tied with a rubber band and a leak of urine at the tied shaft was found. The patient had a history of mental retardation. On genitalia examination, we found that the penis was partially amputated at the proximal shaft of the penis. The urethral anastomosis and penis replantation microsurgery for penis reconstruction were performed. This case highlights the management of traumatic penile partial amputation. On the follow-up after the surgery, the patient had a good wound condition and normal activity with a urinary catheter.

## 3 Surgical procedure

**Fig. 2:**
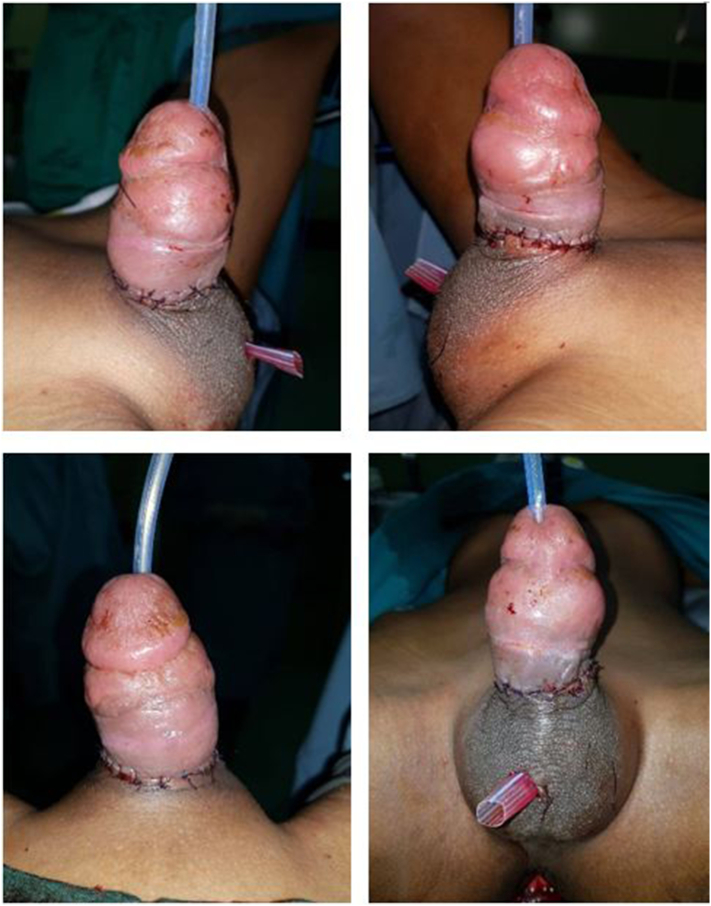
Surgical procedure of urethra resection and penile replantation.

The procedure was performed by a reconstructive urologist under general anesthesia. On physical examination, the distal region from the laceration site was still in vital condition. Debridement of the necrotic tissue was performed at the laceration site on the proximal penile shaft area. The procedure of urethral anastomosis was performed on the partial rupture urethral, ±1 cm in size, at pars pendularae. The urethral anastomosis and penile replantation were successfully performed (Fig. 2). A urinary catheter and drainage were installed. The catheter was maintained for 30 days post-operation. The post-operative treatment was ketoprofen suppository three times a day, pethidine 75 mg on 500 cc Tutofusin drip at 20 rpm for pain management, ranitidine 50 mg IV twice a day, and ceftriaxone 2 g IV once a day.

## 4 Clinical discussion

Penile amputation is a rare urologic emergency. However, it carries major functional and psychological consequences regarding the patient's overall quality of life. Male is prone to have external genitalia trauma more frequently than female because the male is more exposed to violence or extreme exercise [5]. The classification of trauma is important to establish a strategy of treatment. The classification of external trauma in a male could be established by the nature of injury mechanism or anatomic site: accidental versus self-mutilation injury and penis versus penis plus scrotum or perineum [1]. Traumatic injury to the penis may concomitantly involve the urethral [6]. Most of the cases reported with self-mutilation result from severe substance-induced psychosis or underlying psychiatric disorder [7]. In our case, a young male with mental retardation got penile amputation caused by a rubber band [6].

Outcome measures for successful penile replantation have been widely varying, limiting the ability to define a successful penile replantation of an amputated penis clearly [8]. Numerous factors that contribute to the successful penile replantation include the severity of the penile injury or amputation, type and mechanism of injury, team expertise available, duration of ischemia time, and use of a microscope at the time of neurovascular bundle repair [9]. Previous studies suggested that total ischemic time of the penis below 15 h (mean 7 h) is associated with the successful outcome of the penile replantation. The majority of cases have reported primary closure of the urethral and corporal bodies. Previously published data have recommended a temporary placement of a suprapubic catheter at the time of penile replantation for urinary diversion purposes. The most common complications reported were skin necrosis, decreased penile skin sensation, and erectile dysfunction [10], [11].

More distal penile injuries are more technically difficult to repair, particularly with vascular anastomosis due to smaller vessels [1]. A consensus in the contemporary literature acknowledges that the microsurgical revascularization and approximation of the penile shaft structures provide early and adequate restoration of penile blood flow with the best outcome of penile replantation survival, erectile and voiding functions [11], [12], [13].

## 5 Conclusion

A traumatic penile partial amputation is a rare urologic emergency. Self-inflicted amputation is often found in a patient with a history of psychological or mental illness. The limited data on detailed best surgical measures and outcomes is still a concern. Successful penile replantation is commonly measured by restoring intact penile sensation, recovering erectile function, and/or absence of urethral strictures or urinary problems. The primary goals for successful penile replantation are to minimize ischemia time, proper transport of distal penile segment, and transportation to a hospital with the surgical expertise and equipment for the patient's best outcomes.

## Source of funding

This research did not receive any specific grant from funding agencies in the public, commercial, or not-for-profit sectors.

## Consent

Written informed consent was obtained from the patient for publication of this case report and accompanying images. A copy of the written consent is available for review by the Editor-in-Chief of this journal on request.

## Guarantor

Jason Liarto

## Provenance and peer review

Not commissioned, externally peer-reviewed.

## Source of support

Any grants/equipment/drugs, and/or other support that facilitated the conduct of research/writing of the manuscript (including AFMRC project details, if applicable).

## Ethical approval

Ethical clearance was not necessary as the regulation of Hasan Sadikin General Hospital ethical committee

## CRediT authorship contribution statement

Jason Liarto: study concept or design, data analysis or interpretation, writing the paper.

Kuncoro Adi: Data collection, writing the paper.

## Research registration (for case reports detailing a new surgical technique or new equipment/technology)

NA.

## Declaration of competing interest

The authors have no conflict of interest.
